# Occupational accidents involving exposure to biological material reported at a worker’s health sentinel unit: 11,645 cases

**DOI:** 10.47626/1679-4435-2022-699

**Published:** 2023-02-03

**Authors:** Nathália Nakase Mizoguti, Michely Mika Hirota, Fernanda Yuki Ito, Maryna Rodrigues Gonçalves, Mayara Rodrigues Gonçalves, Mariana Rie Hayashida, Edevar Daniel, Paulo Roberto Zétola

**Affiliations:** 1 Medicina, Universidade Federal do Paraná (UFPR), Curitiba, PR, Brazil; 2 Medicina do Trabalho, UFPR, Curitiba PR, Brazil

**Keywords:** occupational accidents, biological materials, epidemiology, acidentes de trabalho, material biológico, epidemiologia

## Abstract

**Introduction:**

Health professionals are exposed to various occupational biological, chemical, physical, and ergonomic risks, in addition to the risk of accidents. Understanding occupational accidents involving biological material in a specific area could be an initial step towards prioritizing correct working conditions.

**Objectives:**

To determine the profile of occupational accidents involving exposure to biological material based on data from a sentinel unit in Curitiba, Brazil.

**Methods:**

This observational, retrospective, descriptive study with quantitative methods collected disease notification system data from 2008-2018.

**Results:**

During the study period, 11,645 cases of occupational accidents involving biological material were reported. Most victims were women (80.4%) and nursing technicians (30.9%). The highest number of accidents involved material found on the floor (11.1%). Regarding personal protective equipment, 69% of the victims used procedure gloves. The years with the most reported accidents were 2016 and 2018. Treatment abandonment was high (56%).

**Conclusions:**

The number of accidents involving biological material was high, as was that of victims who abandoned serological follow-up. Prevention and awareness strategies are necessary to change this scenario.

## Abstract (pt)

**Introdução:**

Na área da saúde, os profissionais estão expostos a variados riscos ocupacionais: biológico, químico, físico, ergonômico e de acidentes. O conhecimento acerca dos acidentes de trabalho com material biológico de determinado setor pode ser o passo inicial para a priorização de medidas de correção das condições de trabalho.

**Objetivos:**

Traçar um perfil dos acidentes de trabalho com exposição a material biológico, com base em dados de uma unidade sentinela de Curitiba, Paraná.

**Métodos:**

Estudo observacional, retrospectivo e descritivo com abordagem quantitativa, realizado através de levantamento de dados do Sistema de Informação de Agravos de Notificação, entre 2008 e 2018.

**Resultados:**

Durante o período, foram notificados 11.645 casos de acidentes de trabalho com exposição a material biológico. A maioria ocorreu em pessoas do sexo feminino (80,4%), e a ocupação mais envolvida foi técnico de enfermagem (30,9%). O maior número de acidentes ocorreu com materiais encontrados no chão (11,1%). Quanto ao uso de equipamentos proteção individual, 69% utilizavam luvas de procedimento. Os anos com maior número de notificações foram 2016 e 2018. Na evolução, houve alta taxa de abandono de tratamento (56%).

**Conclusões:**

O número de acidentes com material biológico é alto, assim como de profissionais que abandonam seguimento sorológico. Estratégias de prevenção e conscientização são necessárias para mudança desse cenário.

## INTRODUCTION

Occupational accidents are an important health problem, particularly for health professionals, who are exposed to risks of a biological, chemical, physical, and ergonomic nature, in addition to accidents. Biological risk entails possible pathogen transmission through contact with contaminated fluids or blood.

Some of the main pathogens in accidents involving biological material are hepatitis B and C and the human immunodeficiency virus, which can have serious consequences for these workers physically, socially, and psychologically.^1^ The U.S. Centers for Disease Control and Prevention estimate that 384,325 percutaneous accidents occur per year among health workers in hospitals,^2^ and these accidents are considered a matter of national and international relevance due to the damage they causes to health workers, employers, and government agencies.^3^

The Brazilian Ministry of Health has developed protocols to care for professionals exposed to biological material, establishing flows of treatment and case notification, requiring health services to have clear written protocols regarding this type of accident. On April 28, 2004, Ordinance 777/MS was passed, which added 11 work-related injuries to the disease notification system (SINAN-NET), including accidents involving biological material.^4^

Although such accidents are frequent in Brazil, neither their number nor their consequences have been adequately determined. The lack of systematized data makes it difficult to plan and implement preventive measures.^5^ Discovering the details of occupational accidents involving biological material in a given geographic area can be an initial step towards correct working conditions.^4^

Thus, we conducted this study to further our understanding of the situation of workers in metropolitan Curitiba in order to help expand the planning, development, and improvement of preventive actions. Its objective was to analyze the distribution of occupational accidents involving exposure to biological material from reports logged in the SINAN-NET database of a sentinel unit in Curitiba, Paraná. A profile of the injured professionals was determined, based on sex, age group, occupation, accident circumstances, personal protective equipment (PPE) use, and accident outcome (discharge with or without serological conversion, discharge because the source patient was serologically negative, and treatment abandonment).

## METHODS

This observational, retrospective, descriptive study with quantitative methods collected data about accidents involving biological material that occurred between 2008 and 2018 and were reported to SINAN-NET by the occupational health unit of the Hospital do Trabalhador in Curitiba, Paraná, Brazil. The circumstances of the accident, the annual number of accidents, and the outcome of the case (left blank, discharge with or without serological conversion, discharge because the source patient was serologically negative, and treatment abandonment) were analyzed. The data analysis consisted of simple descriptive statistics and frequencies, with the data presented in tables and graphs.

Regarding ethical aspects, since this study was based on secondary data from a database, there was no need for research ethics committee approval according to National Health Council Resolution 466/2012 and international ethical guidelines. The authors declare no conflicts of interest.

## RESULTS

Over a period of 11 years (2008-2018), 11,645 accidents involving biological materials were registered in SINAN-NET by the occupational health unit. Table 1 shows the absolute (AF) and relative (RF) frequency of cases according to patient sex, including the predominance of female victims (80.4%). The AF and RF of the cases were also assessed by year, with the most reports occurring in 2016 (FA = 1,351, FR = 11.6%), 2017 (FA = 1,266, FR = 10.9%) and 2018 (FA = 1,358, FR = 11.6%).

**Table 1:** Distribution of accidents involving biological material by sex

Year	Women		Men		Total	
	n	%	n	%	n	%
2008	753	6.50	170	1.50	923	8.00
2009	780	6.70	166	1.40	946	8.10
2010	718	6.20	150	1.30	868	7.50
2011	408	3.50	67	0.60	475	4.10
2012	1010	8.70	200	1.70	1210	10.40
2013	1131	9.70	271	2.30	1402	12.00
2014	900	7.70	251	2.20	1151	9.90
2015	565	4.80	130	1.10	695	5.90
2016	1066	9.20	285	2.40	1351	11.60
2017	1000	8.60	266	2.30	1266	10.90
2018	1029	8.80	329	2.80	1358	11.60
Total	9360	80.40	2285	19.60	11,645	100.00

Source: Disease Notification System (SINAN-NET) data January 2008 to December 2018.

However, of the 11,649 registered cases, only 7165 patients began follow-up at the service. Among those who did, the most common occupations were nursing technicians (30.9%) and nursing assistants (12.1%)(Table 2). The age group with the highest number of accidents was 20-34 years (52.61%), followed by 35-49 years (35.06%).

**Table 2:** Distribution of accidents involving exposure to biological material according to occupation and age group

Occupation (%)			Age range (%)					
			< 20 years	20-34 years	35-49 years	50-64 years	65-79 years	> 80 years
1	Nursing technician	30.90	0.20	15.18	13.10	2.43	0.02	0.00
2	Nursing assistant	12.10	0.10	4.77	5.30	1.90	0.00	0.00
3	Student	8.50	1.00	6.90	0.53	0.00	0.02	0.00
4	Nurse	7.50	0.04	4.80	2.20	0.40	0.02	0.02
5	Refuse collector	3.60	0.22	2.60	0.53	0.20	0.00	0.00
6	Dental Surgeon - General Practitioner	3.50	0.04	1.37	1.44	0.62	0.00	0.00
7	Cleaning staff	3.10	0.04	0.70	1.60	0.78	0.02	0.00
8	Clinic physician	2.60	0.00	2.00	0.44	0.18	0.04	0.00
9	Domestic servant in general services	2.10	0.04	0.53	1.10	0.46	0.00	0.00
10	Building custodian	1.70	0.00	0.44	0.88	0.42	0.00	0.00
Other		24.40	0.80	13.32	7.94	2.23	0.09	0.00
Total		100.00	2.48	52.61	35.06	9.62	0.21	0.02

Source: Disease Notification System (SINAN-NET) data from January 2008 to December 2018.

Regarding the circumstances of the accidents, the main cause was inadequate sharps disposal in the trash (10.5%) or on the floor (11.1%) (Table 3). Regarding PPE use, 72% were not wearing a mask and 74.2% were not wearing protective eyewear at the time of the accident, although 69% were using procedure gloves (Table 4).

**Table 3:** Distribution of accident circumstances

Circumstances of the accident	n	%
Improper sharps disposal		
Trash	753	10.50
Floor	796	11.10
Laundry	80	1.10
Sterilization unit	439	6.10
Handling sharps boxes	548	7.60
Procedure		
Surgical	683	9.50
Dental	707	10.00
Laboratory	266	3.70
Glycemia testing	520	7.30
Re-capping sharps	151	2.10
Other	2222	31.00
Total	7165	100.00

Source: Disease Notification System (SINAN-NET) data from January 2008 to December 2018.

**Table 4:** Use of personal protective equipment at the time of the accident

Type	Unknown^*^	Yes	No
	%	%	%
Apron	18.00	31.20	50.80
Boots	19.30	14.70	66.00
Gloves	12.40	69.00	18.60
Mask	19.00	9.00	72.00
Goggles	19.00	6.80	74.20
Face protection	19.40	0.60	80.00

Source: Disease Notification System (SINAN-NET) data from January 2008 to December 2018.

* ie, no information provided in the notification form

Regarding outcomes (Table 5), 2184 (RF = 30.47%) patients were discharged without serological conversion and 139 (RF = 1.94%) cases were discharged because the source patient was serologically negative. In most cases, however, the accident victim was lost to follow-up (AF = 4,013, RF = 56.04%). The number of reports with missing data was also significant (AF = 826, RF = 11.55%). There were no reported cases of serological conversion after an accident involving biological material during the study period.

**Table 5:** Outcome of accidents with biological material from 2008 to 2018

Year of notification	Unknown^*^		Discharge with serological conversion		Discharge without serological conversion		Discharge - source patient negative		Abandonment	
	n	%	n	%	n	%	n	%	n	%
2008	1	0.01	0	3.25	233	3.25	0	0.00	396	5.52
2009	0	0.00	0	4.41	316	4.41	0	0.00	321	4.48
2010	41	0.57	0	3.73	267	3.73	0	0.00	271	3.80
2011	76	1.06	0	2.60	186	2.60	1	0.01	45	0.63
2012	102	1.42	0	2.62	188	2.62	0	0.00	546	7.62
2013	46	0.64	0	2.13	153	2.13	1	0.01	429	6.00
2014	18	0.25	0	3.96	284	3.96	1	0.01	492	6.87
2015	43	0.60	0	1.72	123	1.72	21	0.30	315	4.40
2016	40	0.56	0	2.76	198	2.76	51	0.71	631	8.80
2017	52	0.72	0	3.17	227	3.17	64	0.90	555	7.75
2018	410	5.72	0	0.12	9	0.12	0	0.00	12	0.17
Total	829	11.55	0	30.47	2184	30.47	139	1.94	4013	56.04

Source: Disease Notification System (SINAN-NET) data from January 2008 to December 2018.

* ie, no information provided in the notification form.

## DISCUSSION

Our study found that many victims of accidents involving biological material do not begin post-exposure follow-up protocol as recommended by the Ministry of Health. This could indicate a lack of worker knowledge or lack of concern about the severity of these accidents. The high rate of abandoning serological follow-up may also reflect this.^6^

Another obstacle found in this study was underreporting. Without an accurate account of the situation, it is more difficult to develop strategies to make work activities safer. This could also be due to a lack of knowledge about the severity of accidents, mainly those involving biological material.^7^

Regarding distribution by sex, the fact that more accidents happened to women can be explained by the predominance of women in nursing and general services,^4^ which are precisely the occupations in which most accidents involving exposure to biological material occur. Nurses spend a great deal of time in direct patient assistance,^7^ which entails greater exposure to accidents. Students also figured prominently in the reports; although they are not in contact with patients as often as nursing professionals, they have less experience and knowledge.^8^ These considerations, however, do not justify the fact that general service and cleaning professionals were among the most affected occupations.

When analyzing the circumstances of the accidents, inadequate sharps disposal was the most important cause, unlike other studies^6,9,10^ that have reported greater occurrence during procedures. This deserves attention, since it is a cause that can be avoided by improving surveillance and routine disposal of contaminated material.

One strategy for improving professional conduct and avoiding accidents is redirecting health professional behavior to include preventive measures. Some behavior patterns in the everyday actions of professionals can be difficult to forget or avoid. The act of re-capping sharps, for example, was seen as correct conduct a few years ago, but today is highly condemned due to the risk of contamination.^6^ In this study, as in others, re-capping sharps was still one of the main causes of accidents.^6,7,9,10^

The use of adequate PPE has been shown to efficiently reduce the risk of contamination during work,^11^ and, according to Regulatory Norm 32, health institutions must provide PPE to their employees.^12^ Our study, however, found low worker adherence to safety equipment protocols. Gloves were the only equipment used in most situations (just under 70% of the cases). Professionals must be retrained about this issue, and their motives for not using PPE must also be understood. However, in the coming years there should be a trend toward fewer accidents resulting from the non-use of PPE, given that the demand for it has increased more than 1,000-fold since the COVID-19 pandemic began.^13^ During the first quarter of 2020 alone, the United Nations delivered more than 6.4 million gloves, 1.8 million surgical masks, and 1 million gowns worldwide.^14^

## CONCLUSIONS

When analyzing the collected data, we found that some fields were improperly filled in, omitting important details about the accident’s circumstances. Thus, it is essential to review these administrative processes and sensitize professionals to the importance of complete information for a reliable database.

In addition, implementing protocols and flowcharts for adequate health professional care, together with biosafety measures and a policy for procedure and activity review, are essential for reducing the rate of accidents involving biological material.^4^ Prevention strategies must include joint actions established between workers and management, which must be aimed at improving working conditions, especially work planning and organization, the provision of safety devices, the implementation of educational programs, and changing worker behavior.^3^ Such strategies must also be adapted to different professional categories and the peculiarities of each job function for efficient prevention.

In addition to taking standard precautions as a preventive measure, using safety devices, such as needleless systems, retractable needles, and needle guard systems, can also help with prevention. Although the North American literature has already shown the positive impact of these devices for reducing sharps injuries, not all health institutions in Brazil invest in this type of equipment.^15,16^

Although according to Regulatory Norm 32, health institutions must provide PPE to their employees,^12,17^ we found low adherence to PPE use by the accident victims. However, due to the COVID-19 pandemic and increased demand for PPE, this is expected to change, contributing to fewer accidents.^13,18^
